# Identification and Characterization of a Polyethylene-Degrading Fungus *Aspergillus sydowii* Isolated from Soils of Waste Disposal Sites

**DOI:** 10.3390/molecules31101557

**Published:** 2026-05-07

**Authors:** Qingyue Wang, Linlu Wang, Xiaoyu Chen, Aozhuo Wang, Youxi Zhao

**Affiliations:** Biochemical Engineering College, Beijing Key Lab Biomass Waste Resource Utilization, Beijing Union University, Beijing 100023, China; wqy190780229@163.com (Q.W.); 15028077759@163.com (L.W.); 19153683503@163.com (X.C.); wangaz0425@163.com (A.W.)

**Keywords:** low-density polyethylene, plastic biodegradation, *Aspergillus sydowii*, laccase, genome sequencing

## Abstract

Petrochemical-based plastics are widely used due to their convenience and low cost, with polyethylene (PE) being the most produced globally. However, the lack of efficient and sustainable treatment methods for conventional plastic wastes has led to severe environmental pollution. A new fungus strain capable of degrading PE was isolated from soil samples collected at a waste disposal site in Henan province and identified as *Aspergillus sydowii* W144. After 30 days of incubation under solid-state culture conditions, the strain demonstrated significant oxidative depolymerization of low-density polyethylene (LDPE). FTIR results revealed a substantial increase in the carbonyl index of the LDPE film, while differential scanning calorimetry (DSC) analysis detected an enhanced crystallinity in the LDPE film. Notably, distinct pitting and erosion marks were observed on the surface of LDPE film using scanning electron microscopy (SEM). Quantitative analysis showed a weight loss rate of 6.39% and a reduction in Weight-Average Molecular Weight (Mw) by 50.93%. Among currently identified PE-degrading strains polyethylene, *A. sydowii* W144 exhibits particularly outstanding depolymerization efficiency, especially on untreated PE. Based on the whole-genome data of *A. sydowii* W144, a preliminary model of the putative polyethylene degradation pathway in *A. sydowii* W144 was constructed through homology-based sequence analysis and by referencing previously reported polyethylene degradation pathways. Laccase/multicopper oxidase plays a key role in the initial oxidation of PE. Heterologous expression of the candidate gene laccase4 in *Pichia pastoris* yielded an active enzyme (~56 kDa) with a laccase activity of 460 U/L, confirming its functionality. This study provides a novel microbial resource and potential enzymatic tools for PE biodegradation. The strain exhibits a promising application in complex ecosystems for PE pollution. IMPORTANCE: The polyethylene-degrading strain *A. sydowii* W144 isolated in this study exhibits highly efficient depolymerization capabilities, particularly under solid-state culture conditions. Genomic sequencing analysis enabled the construction of a potential polyethylene (PE) degradation pathway and facilitated the identification of key laccase and multicopper oxidase genes involved in this process. The isolation of this novel strain enriches the microbial resources available for PE waste treatment and offers new insights into the mechanisms of plastic biodegradation.

## 1. Introduction

Petrochemical-based plastics are renowned for their superior material properties, easy for processing, and cost-effectiveness [1]. Data from Plastics Europe (the only European trade association headquartered in Brussels with representatives across all 27 EU member states) reveals a consistent upward trajectory in global annual plastic production, which rose from 370.7 million tonnes in 2018 to 413.8 million tonnes in 2023. Notably, petroleum-based plastics constitute 90.4% of this total volume [2]. These materials have significantly enhanced modern convenience and generated substantial economic benefits.

However, due to their massive consumption, the disposal of petroleum-based plastic waste has garnered widespread global attention. Hampered by low economic value and the absence of mature recycling infrastructures, the global plastic recycling rate remains persistently low [2,3,4]. Consequently, vast quantities of plastic waste enter the environment through landfilling, incineration, or improper disposal, continuously accumulating in soil and water bodies. This accumulation poses severe threats to both ecosystems and human health [4].

The development of high-efficiency, cost-effective, and sustainable methods for plastic waste treatment has become a priority research area [5]. Among various approaches, microbial degradation offers distinct advantages over physical recycling and chemical degradation. Specifically, it is characterized by superior environmental compatibility, high sustainability, low energy consumption, and the absence of toxic byproducts [6]. Consequently, biological treatment strategies have emerged as a central focus in current research.

Polyethylene (PE), the most widely used petrochemical-based plastic, is particularly recalcitrant due to its high molecular weight and hydrophobic nature [7]. Consequently, extensive studies have tried to isolate PE-degrading microorganisms and investigate the mechanisms underlying microbial PE degradation as a sustainable solution for PE-waste treatment.

As PE is highly hydrophobic, degradation initiates with microbial attachment: bacteria form biofilms, while filamentous fungi use hyphal to adhere to the surface [8,9]. The subsequent biodegradation involves four steps. First, extracellular enzymes (e.g., laccase, manganese peroxidase, and monooxygenase) mediate oxidative depolymerization, introducing polar groups (e.g., =CO) and shortening polymer chains [2,10,11]. Second, intermediates undergo enzymatic hydroxylation. Third, hydroxylated alkanes are converted into fatty acids either via terminal oxidation (involving dehydrogenases) or subterminal oxidation mediated by Bayer–Villiger monooxygenases (BVMOs) followed by esterase hydrolysis [12,13]. Finally, fatty acids undergo β-oxidation to form acetyl-CoA, entering the Tricarboxylic Acid (TCA) cycle for complete mineralization [14].

Although numerous microbial strains, including *Aspergillus sydowii*, capable of adhering to PE, have been isolated from diverse environments (such as marine ecosystems, landfills, wastewater treatment plants, and areas severely affected by plastic pollution), the majority exhibit negligible ability to mineralize high-molecular-weight PE chains [15,16,17,18,19]. Reported degradation rates for pristine PE films are typically extremely low, often resulting in less than 5% weight loss over periods of several months to years without prior physicochemical pre-treatment. Therefore, the isolation and discovery of novel, high-efficiency PE-degrading microorganisms and enzymes remains a critical priority.

In this study, a total of fifteen PE-degrading strains were isolated from landfill soil, with the fungus *A*. *sydowii* W144 selected as the primary candidate due to its rapid growth on PE powder. Following phylogenetic identification and culture optimization, PE degradation by *A. sydowii* W144 was characterized via SEM, FTIR, HT-GPC, and DSC, revealing significant changes in surface morphology, functional groups, molecular weight, and crystallinity of PE film. Then, whole-genome sequencing and analysis enabled the reconstruction of a putative degradation pathway and the identification of encoded enzymes implicated in this process. This work expands the diversity of PE-degrading microbes and provides critical insights for developing effective bioremediation strategies for plastic pollution.

## 2. Results

### 2.1. Isolation and Identification of PE-Degrading Strains

**Figure 1:**
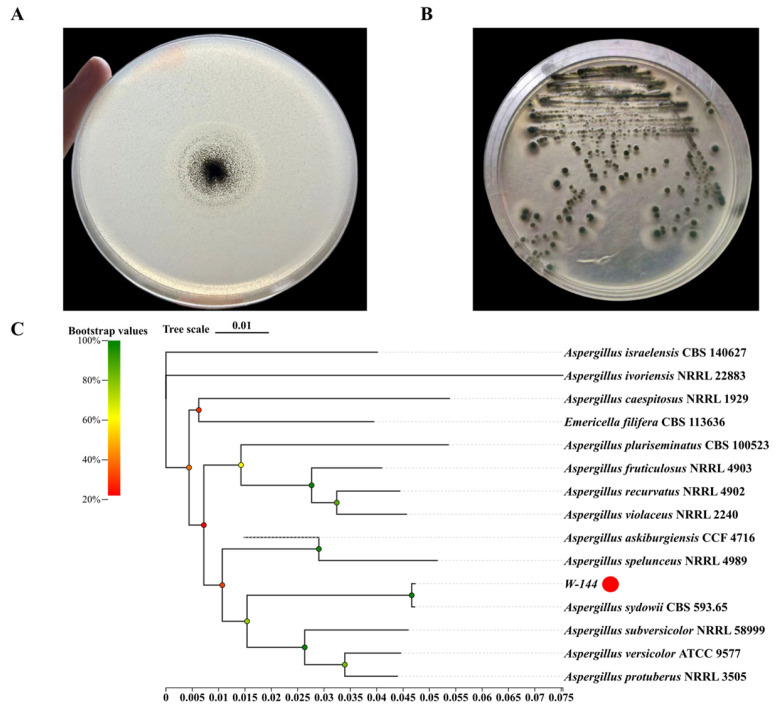
Isolation and phylogenetic analysis of strain W144 using ITS and *beta tubulin* gene (obtained from whole-genome sequencing) sequences. (**A**) Degradation zone (clear zone) produced by strain W144 on MSM agar medium containing LDPE powder as the sole carbon source after three days of incubation at 30 °C. (**B**) Colony morphology of strain W144 grown on PDA medium at 30 °C for three days. (**C**) Phylogenetic tree of strain W144 and related taxa based on ITS sequences. The tree was constructed using the Neighbor-joining method. Node values represent bootstrap support from 1000 replicates (%). The red symbol indicates strain W144. The ITS sequence accession numbers of stains are shown in the parentheses.

Fifteen microbial strains capable of degrading and utilizing PE as the sole carbon source were isolated from the soil samples collected at a plastic waste landfill. To determine their taxonomic status, the 16S rRNA gene (for bacteria) and the ITS region (for fungi) were amplified via PCR and subsequently sequenced. Phylogenetic identification of these strains was conducted by aligning sequences against standard databases, with detailed results summarized in Table S1. Among the isolates, the fungal W144 strain exhibited the most significant PE degradation activity, visually evidenced by the formation of a distinct clear zone surrounding the colonies on PE-containing agar plates (Figure 1A). The W144 colonies displayed characteristic morphological features, including extensive mycelial growth, a distinctive blue pigmentation, and abundant conidial spores on the surface (Figure 1B). BLAST analysis of the W144 ITS sequence (deposited in the NMDC database under accession number NMDCN000A42U) and the *beta-tubulin* gene sequence against the NCBI nucleotide database revealed 100% identity (with 96% query coverage) to the reference strain *Aspergillus sydowii* CBS 593.65 (Figure 1C). Consequently, strain W144 was identified as *Aspergillus sydowii* W144.

### 2.2. Impact of Culture Media Composition on PE Degradation by A. sydowii W144

**Figure 2:**
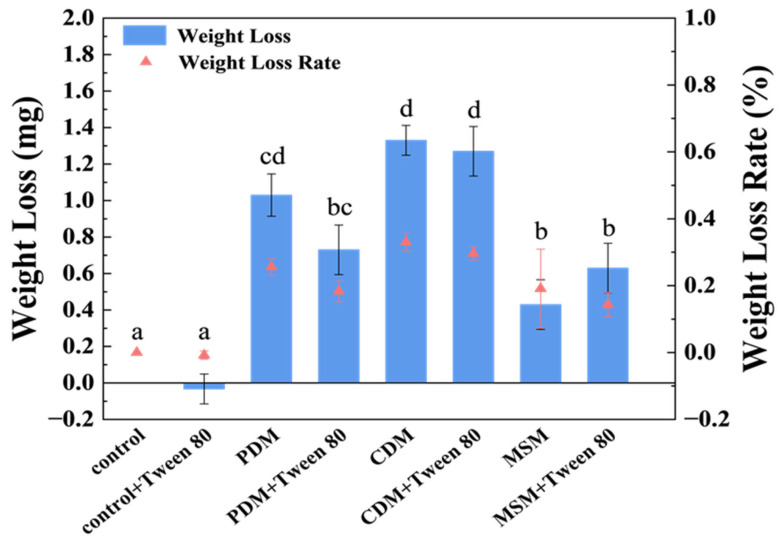
Weight loss of LDPE films degraded by strain W144 under different nutrient conditions for 30 days at 30 °C. A total of 0.4 g LDPE film was added to 100 mL liquid PDM, CDM or MSM with or without Tween 80 (1%, *v*/*v*) for degradation. Control represents the MSM containing LDPE films without inoculation of W144. Each group was performed in triplicate, and data are presented as mean ± SD. Different letters above the bars (e.g., a, b, c, d) indicate significant differences (*p* < 0.05) in weight loss, while bars sharing the same letter indicate no significant difference.

**Figure 3:**
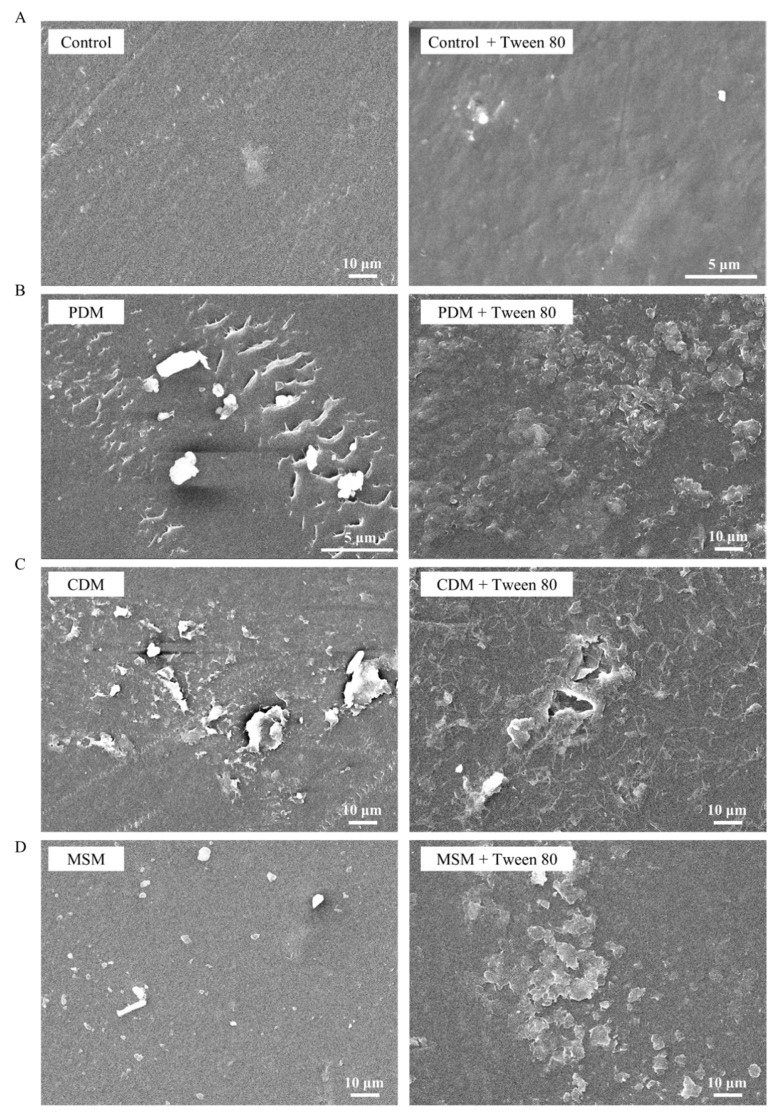
SEM images illustrating the surface deterioration of LDPE films after 30 days of incubation with strain W144 under different nutrient conditions. (**A**) Controls (without W144 inoculation; Left, MSM; right, MSM + Tween 80). (**B**–**D**) Different media: (**B**) Left, PDM; Right, PDM + Tween 80; (**C**) Left, CDM; Right, CDM + Tween 80; (**D**) Left, MSM; Right, MSM + Tween 80. Scale bars represent 10 µm or 5 µm. 0.4 g of LDPE film was added to 100 mL liquid medium with or without Tween 80 (1%, *v*/*v*) for degradation. The experiment was conducted three times, and representative results were shown.

The strain *A. sydowii* W144 was cultured on PDA medium at 30 °C until the late stationary phase, characterized by mature mycelial networks and abundant sporulation. Spores were harvested and suspended in sterile saline to prepare a standardized inoculum with an optical density (OD_600_) of 0.28. This spore suspension was pre-cultured in PDM for 2 days before being inoculated into the liquid degradation media. Degradation experiments were conducted in three distinct liquid media (PDM, CDM, and MSM) to evaluate the influence of nutrient availability on PE degradation. Low-density polyethylene (LDPE) films were immersed in these media, and the cultures were incubated at 30 °C with shaking for 30 days. To investigate the potential enhancement of bioavailability, the non-ionic surfactant Tween 80 was added into. Following the incubation period, the mass loss of LDPE films was gravimetrically determined. As illustrated in Figure 2, the CDM yielded the most significant degradation, with a maximum mass loss of 1.330 ± 0.082 mg, corresponding to a degradation rate of 0.330 ± 0.027%. In comparison, degradation efficacy was lower. Both the nutrient-rich PDM and the minimal MSM exhibited substantially lower degradation efficacy, with the degradation rate ranging from 0.143 to 0.256%. The results suggested that the specific nutrient balance in CDM optimally supports the expression of PE-degrading enzymes. Meanwhile, surface morphological changes in the treated LDPE films were examined using SEM. Consistent with the gravimetric data, LDPE films recovered from the CDM treatment displayed extensive surface deterioration, characterized by deep pitting, cracks, and cavitation. These features indicate more aggressive microbial colonization and enzymatic attack in CDM. In contrast, LDPE films from the PDM group showed moderate surface roughness, while those from the MSM group nearly retained their original surface morphology (Figure 3). The addition of Tween 80 resulted in negligible improvements in mass loss or surface erosion across all media, indicating that under these experimental conditions, surfactant-mediated emulsification did not enhance the biodegradation of LDPE by this strain.

### 2.3. Effect of Culture Media Physical State on PE Degradation by A. sydowii W144

**Figure 4:**
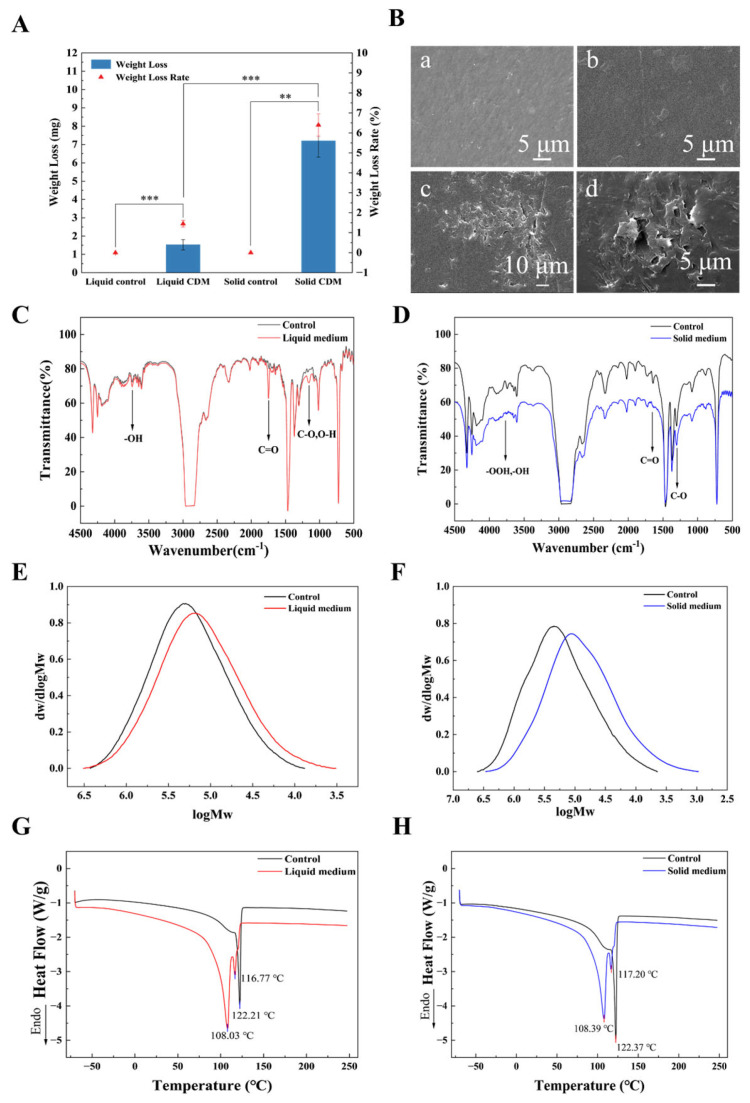
Characterization of LDPE films degraded by strain W144 in liquid and solid CDM media for 30 days at 30 °C. (**A**) Weight loss and degradation rate of LDPE films. Significance levels are indicated as follows: *p* < 0.01 (**), *p* < 0.001 (***). (**B**) SEM images showing surface deterioration and pit formation: (**a**) uninoculated liquid medium (control); (**b**) uninoculated solid medium (control); (**c**) W144-inoculated liquid medium; (**d**) W144-inoculated solid medium. FTIR spectra of LDPE films in (**C**) liquid and (**D**) solid CDM media. Molecular weight of LDPE films degraded in (**E**) liquid and (**F**) solid CDM media. DSC thermograms of LDPE films recovered from (**G**) liquid and (**H**) solid CDM media. All degradation assays were initiated with 0.1 g of LDPE film.

Among the three tested media, strain W144 exhibited the fastest LDPE degradation in CDM. Consequently, further investigation into LDPE degradation by *A. sydowii* W144 was conducted in both liquid and solid CDM media at 30 °C for 30 days. The degraded LDPE films were characterized using weight loss analysis, SEM, FTIR, GPC, and DSC. Degradation in solid CDM resulted in a mass loss rate of 6.39 ± 0.56% (Table 1 and Figure 4A), which was significantly higher than that observed in liquid medium (1.45 ± 0.17%). SEM analysis revealed distinct morphological changes on the degraded films. Compared to the uninoculated controls, the W144-inoculated LDPE surfaces became significantly roughened, exhibiting pitting, cracking, and fragmentation. Notably, the LDPE films degraded in solid CDM displayed more pronounced surface damage (Figure 4B). These morphological observations are consistent with the weight loss data, indicating that LDPE degradation was more extensive in solid CDM.

FTIR analysis (Figure 4C,D) revealed distinct degradation patterns between liquid and solid cultures. In the liquid medium, LDPE films showed a substantial increase in the ester carbonyl peak (1740 cm^−1^) and a slight rise in the ketone peak (1715 cm^−1^). This indicates initial chain scission forming ester groups, which serve as substrates for microbial enzymes (e.g., lipases and esterases) to further hydrolyze the polymer into utilizable small molecules. In contrast, the solid medium group exhibited a significant decrease in overall IR transmittance. This phenomenon, consistent with the severe surface roughening and pitting observed in SEM, likely results from increased light scattering due to morphological damage, alongside chain scission and structural amorphization. Quantitative analysis of the Carbonyl Index (CI) and Double Bond Index (DBI) further confirmed the superiority of solid-state degradation (Table 1). While liquid culture yielded only a slight CI increase and negligible DBI change—likely due to enzyme dilution and limited oxygen transfer—solid culture promoted a ~2-fold increase in both CI and DBI. This enhanced efficiency is attributed to optimal aeration, robust mycelial colonization, and the maintenance of high local enzyme concentrations at the film surface. These conditions facilitate intense, localized oxidation and physical erosion, confirming that solid-state fermentation is more conducive to *A. sydowii* W144-mediated LDPE degradation.

GPC analysis (Figure 4E,F) revealed that degradation by *A. sydowii* W144 shifted the molecular weight distribution of LDPE toward lower values. As summarized in Table 1, Mw decreased by 19.27% in liquid culture and significantly more (50.93%) in solid culture. This substantial reduction in Mw confirms extensive chain scission, particularly under solid-state conditions. Concurrently, the polydispersity index (PDI) increased by 19.70% (liquid) and 25.71% (solid), indicating a broader distribution of fragment sizes as high-molecular-weight chains were broken down. These results demonstrate that while *A. sydowii* W144 effectively degrades LDPE in both media, the solid-state culture promotes significantly more efficient depolymerization, consistent with the observed weight loss and surface erosion.

DSC analysis (Figure 4G,H) revealed that biodegradation significantly altered the thermal properties of LDPE. The second heating curve for the liquid degradation group appeared steeper than that of the solid group, likely due to uneven heat transfer caused by residual surface fragments, as corroborated by SEM observations. While both groups exhibited similar decreases in melting (*Tm*) and crystallization (*Tc*) temperatures, distinct differences emerged in enthalpy and crystallinity (*Xc*).

As detailed in Table 2, *Xc* of LDPE samples increased in both treatments but was more pronounced in the solid medium. The liquid group rose from 30.46% to 38.57%, increasing by 21.0%, whereas the solid group surged from 25.53% to 34.00%, increasing by 33.2%. This increase in crystallinity might be attributed to two concurrent mechanisms: (1) the preferential degradation of amorphous regions, which enriches the relative proportion of crystalline domains, and (2) chain scission reducing molecular weight, thereby enhancing chain mobility and facilitating recrystallization [20,21,22,23,24]. The greater crystallinity increase in the solid group suggests more extensive chain scission and amorphous region removal, consistent with the higher degradation efficiency observed in GPC and FTIR analyses.

### 2.4. Prediction of Key Polyethylene Degrading Enzymes

The complete genome of *A*. *sydowii* W144 (Accession: NMDC20441221) was successfully sequenced and assembled. The genome spans 34,772,622 base pairs with a G+C content of 49.86%. Functional annotation against major databases (NR, KEGG, GO, KOG, CAZy, Pfam, and Swiss-Prot) was carried out to assign the putative functions of the coding genes. Totally, the strain encodes 13,414 protein-coding genes and 371 non-coding RNA genes. Based on the annotation, we predicted the possible PE biodegradation pathways, which involves a complex enzymatic cascade comprising laccases, multicopper oxidases, peroxidases, oxygenases, hydroxylases, oxidoreductases, dehydrogenases, carboxylases, esterases, lipases, and chitinases. Building on established metabolic pathways derived from integrated metabolite and enzyme profiling, we screened the *A. sydowii* W144 genome for homologs of verified PE-degrading enzymes from other *Aspergillus* species. Homology alignment revealed significant matches across key enzyme families (Table S2). Specifically, alignment with the multicopper oxidase from *A. clavatus* identified 13 CDS sequences with over 28% homology, the highest similarity reaching 47.57%. Similarly, comparison with the peroxidase from *A. oryzae* revealed 5 homologous sequences, with a maximum identity of 83.28%. Screening against the hydroxylase from *A. nomiae* returned 28 matches, with the top hit showing 79.31% similarity. Furthermore, extensive homology was observed with the dehydrogenases from *A. nidulans*; the alcohol dehydrogenase matched 40 sequences with a peak similarity of 93.63%, while the aldehyde dehydrogenase aligned with 56 sequences, reaching a maximum identity of 88.26%.

**Figure 5:**
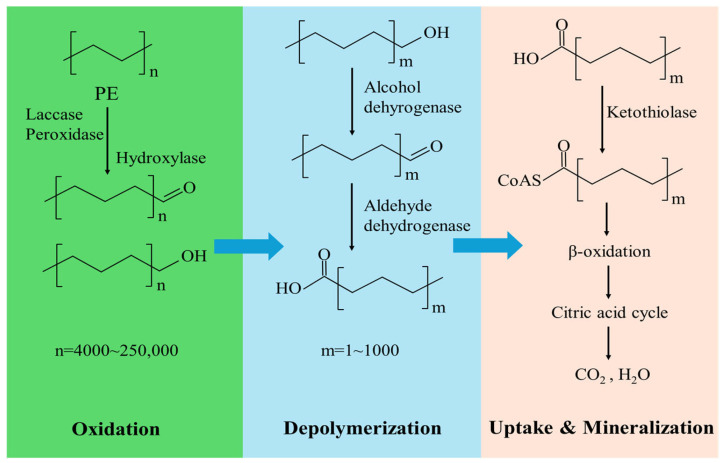
Predicted metabolic pathways involved in PE degradation based on the genome sequence of *A. sydowii* W144. The degradation process comprises three steps: oxidation, depolymerization, and uptake and mineralization. The potential enzymes catalyzing the reactions are summarized in Table S2.

Based on current insights into fungal LDPE degradation mechanisms and these genomic findings, we propose a putative three-stage polyethylene (PE) degradation pathway for *A. sydowii* W144 (Figure 5). In the initial oxidation stage, multicopper oxidases (including laccases), peroxidases, and hydroxylases introduce oxygen-containing functional groups into the inert polymer backbone. This is followed by the depolymerization stage, which is primarily driven by alcohol dehydrogenases and aldehyde dehydrogenases that further oxidize and cleave intermediate fragments. Finally, in the assimilation and mineralization stage, the resulting low-molecular-weight metabolites—specifically carboxylic acids, alcohols, and aldehydes—are funneled into central metabolic routes such as β-oxidation, leading to their complete mineralization.

### 2.5. Validation of a PE-Degrading Laccase from A. sydowii W144

To functionally validate the genomic predictions regarding PE degradation, four candidate laccase-encoding genes—specifically [EVMptg000005lG015730.1, Laccase1], [EVMptg000005lG008460.1, Laccase2], [EVMptg000004lG001590.1, Laccase3], and [EVMptg000009lG000410.1, Laccase4], which exhibited high homology to known fungal laccases—were selected for heterologous expression. Prior to cloning, the coding sequences were codon-optimized to match the bias of *Pichia pastoris*, thereby maximizing translational efficiency. The synthesized genes were individually ligated into the pGAPZ(α)A expression vector under the control of the constitutive GAP promoter. Following linearization, the recombinant plasmids were transformed into *P. pastoris*, allowing for stable integration into the host genome through homologous recombination.

**Figure 6:**
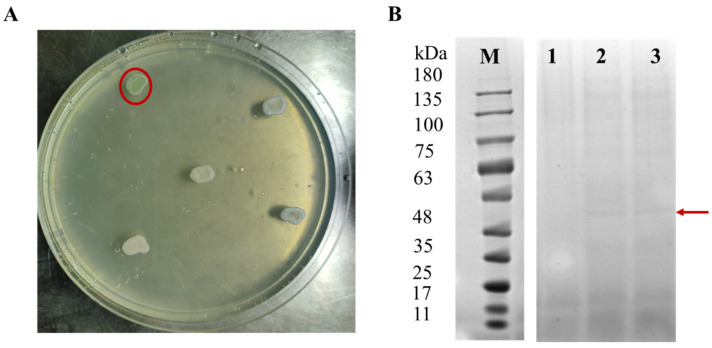
Heterologous expression of Laccase4 from strain W144 in *Pichia pastoris* X33. (**A**) Screening of positive *P. pastoris* transformants harboring the genome-integrated pGAPZ(α)A-LAC-His-linearized plasmid. In positive clones, Laccase4 catalyzes the oxidation of ABTS to its green radical form (ABTS^+^), causing the colonies to exhibit a green phenotype. (indicated by a red circle). (**B**) SDS-PAGE analysis of culture supernatants from positive *P. pastoris* transformants grown in YPD medium. The band indicated by the red arrow indicated the expressed Laccase2 enzyme. Lanes: 1, control of *P. pastoris* (2 days); 2, positive transformant (2 days); 3, positive transformant (3 days); M, protein marker.

Among the four transformants, only the strain harboring Laccase4 demonstrated successful protein secretion and enzymatic activity. This was initially confirmed by a qualitative plate assay, where colonies developed a distinct green oxidation halo on agar containing 2,2′-azino-bis (3-ethylbenzothiazoline-6-sulfonic acid) (ABTS), indicative of typical laccase-mediated substrate oxidation (Figure 6A). The other three candidates failed to show detectable activity, potentially due to issues with protein folding, secretion bottlenecks, or post-translational modifications in the heterologous host.

Subsequent shake-flask cultivation of the positive Laccase4 strain was conducted for 72 h. SDS-PAGE analysis of the cell-free culture supernatant revealed a prominent, Coomassie-stained protein band within the 48–63 kDa range, which was absent in the negative control (Figure 6B). The apparent molecular weight of this recombinant protein was estimated at approximately 56 kDa, consistent with the theoretical mass of glycosylated fungal laccases. Quantitative enzymatic assays further confirmed its functionality; the laccase activity in the supernatant peaked on day 3, reaching 460 U·L^−1^. These results conclusively demonstrate that the identified gene encodes a functional laccase capable of oxidizing recalcitrant substrates, supporting its potential role in the initial oxidative attack on the PE polymer chain.

## 3. Discussion

To tackle the biodegradation resistance of polyethylene (PE), in this work we isolated *A*. *sydowii* W144, a novel fungal strain showing remarkable LDPE-depolymerizing capability. Within 30 days of solid-state cultivation, W144 reduced PE film weight by 6.39% and its average molecular weight by 50.93%. The strain proved effective in both liquid and solid cultures, with significantly enhanced activity observed under solid-state conditions.

Table S3 [9,16,25,26,27,28,29,30,31,32,33,34,35,36,37,38,39,40,41] summarizes the degradation efficiencies of various PE-degrading strains reported in the literature alongside the strain isolated in this study. Reported incubation periods typically range from 30 to 140 days. Due to the recalcitrant nature of PE, most strains exhibit moderate efficiency, with weight loss generally remaining below 7%. While some studies report higher values, these often involve specific conditions or substrates. For instance, *Aspergillus niger*/*flavus*/*oryzae* achieved a 26.15% weight loss of LDPE film over 55 days [36]. Notably, *Hypocrea lixii* reportedly degraded PE by 60% within 35 days [38]; however, this was achieved using water-leached LDPE milk packaging fragments subjected to thermal pretreatment (50 °C for 5 days). Similarly, *Aspergillus niger* TA3, isolated from landfill soil, yielded a 46.57% weight loss after 45 days using commercial LDPE bags (25–40 µm thick) [39]. These findings align with repeated reports that pretreated PE is significantly more susceptible to biodegradation. Given the scarcity of strains capable of degrading untreated PE efficiently, the performance of *Aspergillus sydowii* W144 is particularly noteworthy. W144 achieved a 6.39% weight loss of untreated PE film within just 30 days, representing a competitive and relatively high oxidative depolymerization level compared to current benchmarks for pristine plastics. Microbial degradation of PE fundamentally involves cleaving high-molecular-weight chains into lower-molecular-weight fragments; thus, the reduction in molecular weight (Mw) serves as a direct indicator of degradation efficacy. The highest Mw reduction reported to date is 95%, achieved by *Alternaria alternata* FB1(isolated from marine sediment) after 120 days [9]. *Sterigmatomyces halophilus* also demonstrated a substantial 77.9% reduction after 45 days [34]; however, its substrate underwent ethanol sterilization, a process known to alter PE surface properties and facilitate microbial attack. Likewise, *Aspergillus flavus* PEDX3 reduced Mw by 59.5% in 28 days but utilized UV-sterilized HDPE microplastics (<200 µm), where UV irradiation had already compromised the polymer structure [37]. Furthermore, *Bacillus* sp. C2, screened from a long-term landfill, decreased Mw by 51.53% over 60 days using LDPE powder derived from agricultural film [28]; the powdered form inherently enhances microbial adsorption and accelerates degradation. In contrast, *A. sydowii* W144 achieved a 50.93% reduction in Mw within only 30 days using untreated PE film. When considering the absence of physical or chemical pretreatments, this rate of molecular weight reduction positions W144 among the most effective PE-degrading strains currently documented.

The physiological robustness of *Aspergillus sydowii* [42] provides a critical context for evaluating the potential of strain W144 in practical applications. Unlike many laboratory-adapted strains that struggle under fluctuating conditions, the inherent stress regulation mechanisms of *A. sydowii* endow W144 with a unique capacity to survive and function in complex, heterogeneous milieus. This adaptability addresses a major bottleneck in plastic biodegradation: the gap between efficacy under controlled laboratory conditions and practical utility. In light of biosafety considerations, we advocate for strictly confining the application of this strain to artificially controlled environments, such as microplastic-enriched wastewater treatment systems, closed bioreactors, or fermentation tanks. Furthermore, to further mitigate potential risks, future strategies will prioritize metabolic engineering approaches, such as knocking out virulence-related genes or transferring key degradative pathways to safer heterologous hosts (e.g., Saccharomyces cerevisiae) for secure and efficient scalable bio-upcycling.

Therefore, the isolation of W144 represents more than just an addition to the microbial library; it offers a resilient biological chassis for future applications. While our phenotypic data confirmed its oxidative depolymerization capacity, the true value of this strain lies in its genomic potential. By shifting the focus from mere observation of weight loss to a genomic dissection of its degradative machinery, this study bridges the gap between phenotypic performance and molecular mechanism. This mechanistic insight is pivotal for the next phase of research: the metabolic engineering of robust fungal strains to accelerate degradation kinetics and enable scalable plastic bio-upcycling.

Moving beyond phenomenological descriptions of PE degradation, our genomic analysis allows us to propose a coherent mechanistic model for how *A. sydowii* W144 assimilates this recalcitrant polymer. The rate-limiting step in PE biodegradation is universally acknowledged as the initial oxidative cleavage of inert C–C bonds [7]. Our findings strongly support the hypothesis that laccases (multicopper oxidases) are the primary drivers of this initiation step in W144. This aligns with comparative studies, such as those by Gao et al. [9], which demonstrated the superior capability of laccases over peroxidases in attacking the hydrophobic PE surface. The identification of a specific repertoire of laccase-encoding genes in the W144 genome suggests a specialized evolutionary adaptation for oxidizing high-molecular-weight polymers. We postulate that these enzymes catalyze the insertion of oxygenated functional groups (e.g., carbonyl and hydroxyl) into the PE backbone, effectively transforming the hydrophobic surface into a hydrophilic substrate. This “bio-priming” event is crucial, as it renders the polymer chains accessible to downstream hydrolases and facilitates microbial uptake of low-molecular-weight fragments. Consequently, the proposed laccase-centric pathway not only explains the observed degradation efficiency of W144 but also establishes a comparative framework for screening other fungal species. Future efforts should prioritize the heterologous expression and kinetic characterization of these specific laccases to validate their direct role and optimize their catalytic efficiency for industrial applications.

Our genomic analysis of *A. sydowii* W144 revealed a robust oxidative potential, identifying 13 laccase homologs (>28% identity). Among these, Laccase4 emerged as the primary functional driver for LDPE oxidation. Heterologous expression in *Pichia pastoris* yielded a ~56 kDa protein with significant activity (460 U·L^−1^), providing definitive evidence that W144 possesses the specific machinery to initiate inert C–C bond cleavage. The exclusivity of this activity highlights Laccase4 as a prime target for biocatalytic applications, distinguishing it within the strain’s diverse enzymatic repertoire. The lack of detectable activity in the remaining three homologs likely reflects biological complexity rather than functional absence. Factors such as improper folding, cofactor limitations, or strict native regulatory requirements may have suppressed their expression in the heterologous host. This suggests that W144’s full degradative power might rely on a synergistic, tightly regulated enzyme network that functions optimally only in its native context, warranting future optimization of expression strategies to unlock this latent potential.

To advance PE degradation from laboratory observation to industrial application, future research should prioritize: (1) integrating multi-omics to decipher dynamic regulatory networks governing enzyme coordination; (2) employing protein engineering to enhance the thermal stability and affinity of key laccases; and (3) constructing synthetic multi-enzyme consortia for efficient cascade reactions. Ultimately, coupling these strategies with metabolic engineering to redirect degradation intermediates toward high-value chemicals will shift the paradigm from simple waste disposal to sustainable carbon valorization.

## 4. Materials and Methods

### 4.1. PE Materials

Low-density polyethylene (LDPE) in both powder and film forms was used in this study. The LDPE powder (density: 0.920 g/cm^3^, particle size: 500 mesh, approx. 25 µm) was purchased from Putian Yaonan Trading Co., Ltd. (Putian, China). The LDPE film (Model: ET31-FM-00025, density: 0.92 g/cm^3^, thickness: 0.025 mm), confirmed to be free from additives, was obtained from Goodfellow (Shanghai) Trading Co., Ltd. (Shanghai, China).

### 4.2. Strain Isolation and Media

Soil samples were collected from a plastic waste landfill (34.133679° N, 112.818521° E). A 10% (*w*/*v*) soil suspension was prepared in sterile water and serially diluted (10^−4^ to 10^−6^). Aliquots (100 µL) were spread onto minimal salt medium (MSM) plates supplemented with PE as the sole carbon source. The medium (g/L) contained: K_2_HPO_4_ 0.3, NaCl 0.5, MgSO_4_·7H_2_O 1.0, FeSO_4_·7H_2_O 0.01, CaCO_3_ 0.1, MnCl_2_·4H_2_O 0.02, ZnSO_4_ 0.07, Agar 20.0 (pH 7.2). After autoclaving at 121 °C for 30 min, PE powder was dusted onto the semi-solidified agar surface. Plates were incubated inverted at 30 °C for 7 days until colonies appeared.

### 4.3. Phylogenetic Trees Construction

Selected colonies on screening plates were inoculated into Luria–Bertani (LB) for bacteria or Potato Dextrose Agar (PDA) for fungi medium and incubated. Genomic DNA was extracted from the cultures and used as a template for amplifying the 16S rRNA gene (for bacteria) or the ITS region (for fungi). Bacterial 16S rRNA genes were amplified using primers 27F and 1492R, while fungal ITS regions were amplified using primers ITS1 and ITS4. The resulting PCR products were sequenced by Tsingke Biotechnology Co., Ltd. (Beijing, China). Phylogenetic trees were constructed based on the 16S rRNA or ITS sequences using MEGA 7.0 software with the Neighbour-joining method, following sequence alignment and homology search against the NCBI database via BLAST (https://blast.ncbi.nlm.nih.gov/Blast.cgi, accessed on 29 April 2026).

### 4.4. LDPE Degradation Assays

Strains were cultured in PDA medium [43] until the stationary phase (characterized by mature mycelium and abundant sporulation), and then spore suspensions were prepared, followed by OD_600_ measurement. For the liquid degradation assay, 10 mL of spore suspension was added to 90 mL of Potato Dextrose Medium (PDM) [36] and incubated for 2 days. Subsequently, the resulting mycelial pellets were harvested by centrifugation, washed three times with 0.9% (*w*/*v*) saline and resuspended in 10 mL of sterile water. This suspension was inoculated into 250 mL flasks containing 90 mL of three liquid media: PDM, Czapek-Dox Medium (CDM), or Minimal Salt Medium (MSM), each supplemented with 0.4 g of LDPE film (cut into 4 cm × 4 cm pieces). To investigate whether Tween 80 promotes degradation, three additional sets were established using the respective media supplemented with 1% (*v*/*v*) Tween 80. Uninoculated controls containing LDPE with or without Tween 80 were established. Cultures were incubated at 30 °C with shaking at 150 rpm for 30 days.

For the solid degradation assay, 0.1 g of LDPE film (cut into 1 cm × 1 cm pieces) was placed evenly on the surface of solid CDM. The spore suspension was spotted onto the agar surface and streaked around the film edges using the entire volume to ensure the same inoculum load as the liquid assay. Plates were incubated upside down at 30 °C for 30 days [44]. Liquid degradation experiments were conducted simultaneously under identical conditions. Uninoculated solid and liquid controls were established for both assays. All experiments were performed in triplicate.

## 5. Analytical Methods

### 5.1. Characterization of Degraded LDPE Films

Weight Loss Determination of LDPE Film: LDPE films were retrieved, rinsed with distilled water, and soaked overnight in 2% (*w*/*v*) sodium dodecyl sulfate (SDS). Subsequently, the films were ultrasonically cleaned in graded ethanol concentrations for 20 min to completely remove biofilms and adherents. After a final rinse with distilled water, the films were dried in an oven at 60 °C until a constant weight was achieved. The percentage of weight loss was calculated using the following equation:

Weight Loss (%) = [(*m*_0_ − *m*_1_)/*m*_0_] × 100%

where *m*_0_ is the initial mass and *m*_1_ is the mass after degradation.

Scanning Electron Microscopy (SEM) Analysis: Small sections of the treated LDPE films were sputter-coated with gold using a Bal-Tec SCD 005 high-vacuum metallizer (Bal-Tec, Balzers, Liechtenstein) under argon discharge (40 mA, 50 s). Surface morphology of coated-films was examined using a JSM-IT500HR scanning electron microscope (JEOL, Tokyo, Japan) at accelerating voltages of 5.0, 10.0, and 15.0 kV. SEM analysis focused on identifying physical degradation features, including microcracks, surface erosion, pits, and cavities.

Fourier Transform Infrared (FTIR) Spectroscopy Analysis: The surface chemical structure of cleaned LDPE films was analyzed using a IRTracer-100 Fourier Transform Infrared spectrometer (Shimadzu, Kyoto, Japan). Spectra were recorded in transmission mode against an air background over the range of 4000–500 cm^−1^ with a resolution of 4 cm^−1^, accumulating 45 scans per sample to assess LDPE degradation preliminarily. Key absorption bands monitored included ester carbonyl (1740 cm^−1^), ketone carbonyl (1715 cm^−1^), internal C=C bond (1640 cm^−1^), terminal vinyl bond (915–905 cm^−1^), and methylene group (1465 cm^−1^). To quantify oxidative degradation, the Carbonyl Index (CI, A_1715_/A_1465_) and Double Bond Index (DBI, A_1640_/A_1465_) were calculated.

Differential Scanning Calorimetry (DSC): Thermal properties were measured using a DSC-4000 system (PerkinElmer, Shelton, CT, USA). Approximately 5 mg of cleaned LDPE samples were sealed in aluminium pans. High-purity nitrogen was used as the purge gas at a flow rate of 50 mL/min. The thermal protocol consisted of: (1) heating from room temperature to 250 °C at 10 °C/min, followed by a 2 min isothermal hold; (2) cooling to −70 °C at 10 °C/min with a 2 min hold; and (3) a second heating scan to 250 °C at 10 °C/min. The crystallization temperature (*Tc*) during cooling and the melting temperature (*Tm*) from the second heating cycle were determined from the resulting thermograms.

High-Temperature Gel Permeation Chromatography (HT-GPC): Molecular weight distribution was analyzed to assess chain scission and degradation extent, providing an accurate data of degradation extent and rate under different conditions. Approximately 8 mg of the cleaned LDPE films were dissolved in 4 mL of 1,2,4-trichlorobenzene (TCB). Analysis was performed on an Agilent PL-GPC 220 integrated HT-GPC system equipped with a refractive index detector and three 300 × 7.5 mm PLgel MIXED-BLS columns (Agilent Technologies, Santa Clara, CA, USA). TCB served as the mobile phase at a flow rate of 1.0 mL/min and a column temperature of 150 °C. The injection volume was 200 µL. Polystyrene powder (PS) with a particle size of 900.00 µm, in the form of white expandable beads containing pentane and exhibiting a density of 0.60000 g/cm^3^, was purchased from Goodfellow (Shanghai) Trading Co., Ltd. (Shanghai, China).

### 5.2. Genome Sequencing and Pathway Analysis

Whole-genome sequencing was conducted by Biomarker Technologies (Beijing, China) using the PacBio Sequel II system. Circular Consensus Sequencing (CCS) reads were de novo assembled using Hifiasm (v0.15), and the resulting assembly was further polished with Pilon (v1.23) using Illumina short-read data to enhance accuracy. Gene structures were predicted by integrating multiple evidence sources with EVM (v1.1.1), followed by manual curation and adjustment using PASA (v2.0.2). Predicted protein sequences were aligned against the NR, Swiss-Prot, TrEMBL, KEGG, and KOG databases using BLAST (v2.10.0) with an E-value threshold of 1 × 10^−5^. Functional annotation was performed using Blast2Go (v5.2.5; BioBam, Valencia, Spain) for Gene Ontology (GO) terms and HMMER (v3.3.2) for Pfam domain identification.

Based on the KEGG pathway database and the gene annotation of strain W144, we analyzed the metabolic pathways involved in PE degradation, following previous reports [9,14,45]. Enzymes from the genus *Aspergillus* with known functions in PE degradation were identified and aligned against the genome of *A. sydowii* W144 to predict potential candidate genes. Candidates exhibiting at least 28% sequence identity were selected for further analysis.

### 5.3. Enzyme Expression

Four *laccase* genes were codon-optimized for expression in *Pichia pastoris* and each gene sequence was synthesized by Qingke Biotechnology Co., Ltd. (Beijing, China). Then, it was cloned into the pGAPZ(α) vector. The expression host employed was *P. pastoris* X33. The recombinant plasmid was linearized with the restriction enzyme *Bsp*HI (Cat# R0517V; New England Biolabs, Ipswich, MA, USA) and purified using the Solarbio^®^ Cat# D1200 Agarose Gel DNA Recovery Kit (Beijing Solarbio Science & Technology Co., Ltd., Beijing, China). Competent yeast cells were prepared, and transformation was performed following the manufacturer’s instructions for the Beyotime^®^ Cat# D0308S Yeast Transformation Kit (Beyotime Biotechnology, Shanghai, China). Transformants were initially screened on YPDS [46] plates supplemented with 100 μg/mL Zeocin. Putative positive colonies were subsequently verified on indicator plates containing 2,2′-azino-bis (3-ethylbenzothiazoline-6-sulfonic acid) (ABTS).

### 5.4. Assay of Laccase Activity

The recombinant *P. pastoris* X33 harbouring the pGAPZ(α)A-LAC-His plasmid was inoculated into Yeast Extract Peptone Dextrose (YPD) [47] medium and incubated at 28 °C with shaking for 3 days. The culture supernatant was collected by centrifugation and analyzed for protein expression via SDS-PAGE. Laccase activity was determined using the 2,2′-azino-bis (3-ethylbenzothiazoline-6-sulfonic acid) (ABTS) oxidation method. The reaction mixture (3.0 mL total volume) contained 2.8 mL of 100 mM sodium acetate buffer (pH 4.5), 0.1 mL of 1 mM ABTS substrate, and 0.1 mL of enzyme solution. Prior to initiation, the buffer and substrate solutions were pre-incubated at 45 °C for 5 min. The reaction was initiated by adding the enzyme solution, and the increase in absorbance at 420 nm (A_420_) was monitored kinetically for 5 min. One unit (U) of laccase activity was defined as the amount of enzyme required to oxidize 1 μmol of ABTS per minute under the assay conditions. Activity was calculated using the following equation:

$$U=\frac{10^{6}\times V_{1}\times ∆A\times N}{V_{2}\times \varepsilon \times ∆t}$$

where ∆*A* is the change in absorbance at 420 nm over the linear range; Δ*t* is the reaction time interval (min); $V_{1}$ is the total reaction volume (mL); $V_{2}$ is the volume of enzyme solution added (mL); ε is the molar extinction coefficient of ABTS at 420 nm (ε_420_ = 36,000 M^−1^·cm^−1^); and N is the dilution factor of the enzyme sample. The factor 10^6^ converts the result from moles to micromoles (µmol).

## 6. Statistical Analysis

All experiments in this study were performed in triplicate, and data are presented as the mean ± standard deviation (SD). Statistical analysis was performed using one-way ANOVA (SPSS version 27.0; IBM Corp., Armonk, NY, USA). A post hoc Tukey’s test (*p* < 0.05) was conducted to determine significant differences between experimental groups for the weight reduction in LDPE films after 30 days of incubation.

## 7. Conclusions

This study successfully isolated 15 potential PE-degrading strains from landfill soil, identifying *A. sydowii* W144 as a standout candidate with remarkable degradative capacity. Through systematic medium optimization, we established that solid-state fermentation in CDM significantly outperforms liquid culture, achieving a 6.39% weight loss of LDPE within 30 days. Mechanistically, this enhanced efficiency in solid-state conditions is attributed to the formation of dense mycelial networks that tightly adhere to the polymer surface, creating localized high-concentration enzyme systems that drive deep erosive oxidative depolymerization, whereas liquid cultures exhibited less intensive action. Genomic mining coupled with functional validation confirmed the critical role of oxidative enzymes, specifically verifying the activity of a key laccase (Laccase4) among four identified candidates. Collectively, these findings underscore *A. sydowii* W144 as a robust biological agent for PE remediation, particularly under solid-state conditions, and provide a validated genetic reservoir for plastic bio-upcycling. Future research will focus on elucidating the synergistic mechanisms of its full enzymatic repertoire and engineering these pathways to develop scalable, sustainable solutions for global plastic pollution.

## Figures and Tables

**Table 1:** Changes in weight loss, molecular weight, aldehyde value, and double bond index of degraded LDPE film.

Samples	Weight Loss Rate (%)	Mn	Mw	Polydispersity (PDI)	Mw Reduction (%)	Carbonyl Index (10^−2^)	Double Bond Index (10^−2^)
Control-1	0.01 ± 0.00	106, 625	287,131	2.69	0.00	2.37 ± 0.03	2.52 ± 0.01
Liquid CDM	1.45 ± 0.17	71, 897	231, 791	3.22	19.27	4.46 ± 0.09	2.96 ± 0.05
Control-2	0.00 ± 0.00	85, 654	330, 084	3.85	0.00	2.99 ± 0.48	3.34 ± 0.24
Solid CDM	6.39 ± 0.56	33, 497	161, 968	4.84	50.93	9.34 ± 2.87	10.06 ± 3.09

Control-1: the LDPE film degraded in liquid CDM, without the inoculation of *A. sydowii* W144. Control-2: the LDPE film degraded in solid CDM, without the inoculation of *A. sydowii* W144. Mn: Number-Average Molecular Weight. Mw: Weight-Average Molecular Weight.

**Table 2:** Changes in the crystallinity of degraded LDPE films degraded in CDM liquid or solid medium.

Samples	Δ*Hm* (J/g)	Crystallinity *Xc* (%)	Increase in Crystallinity (%)
Control-1	89.25	30.46	
Liquid CDM	113.01	38.57	21.02
Control-2	74.82	25.54	
Solid CDM	99.63	34.00	33.16

Control-1: the LDPE film degraded in liquid CDM, without the inoculation of *A. sydowii* W144. Control-2: the LDPE film degraded in solid CDM, without the inoculation of *A. sydowii* W144. The melting enthalpy (Δ*Hm*) was obtained by integrating the melting peak from the DSC thermogram. The degree of crystallinity (*Xc*) was calculated using the following equation: *Xc* = Δ*Hm*/Δ*Hf*, where Δ*Hm* represents the measured melting enthalpy of the sample (J/g) and Δ*Hf* is the melting enthalpy of 100% crystalline polyethylene, taken as 293 J/g.

## Data Availability

The original contributions presented in this study are included in the article. Further inquiries can be directed to the corresponding author.

## Supplementary Materials

The following supporting information can be downloaded at: https://www.mdpi.com/article/10.3390/molecules31101557/s1.Table S1: List of PE-degrading strains isolated from Henan landfill sites in this study, Table S2: Predicted genes involved in PE degradation pathway in *A. sydowii* W144 based on its genome sequence, Table S3: Summary of reported PE-degrading microorganisms and their degradation characteristics.
