# Supplementary material for: Identification and Characterization of a Polyethylene-Degrading Fungus Aspergillus sydowii Isolated from Soils of Waste Disposal Sites
Source: Molecules. 2026 May 7;31(10):1557. doi: 10.3390/molecules31101557 (PMC13209233; doi:10.3390/molecules31101557)
Supplement: Supplementary file 1 [file molecules-31-01557-s001.zip › molecules-4245209-supplementary.pdf]

Supplementary Information

Identification and Characterization of a Polyethylene-Degrading Fungus *Aspergillus sydowii* Isolated from Soils of Waste Disposal Sites

Qingyue Wang[a], Linlu Wang[a], Xiaoyu Chen[a], Aozhuo Wang[a], Youxi Zhao[a,*]

[a]*Biochemical Engineering College, Beijing Key Lab Biomass Waste Resource Utilization, Beijing Union University, Beijing 100023, PR China*

*Correspondence: Youxi Zhao (Email: zhaoyouxi@buu.edu.cn)

Tables

Table S1. List of PE-degrading strains isolated from Henan landfill sites in this study.

SN.	Category	Top-hit strain	Sequence Similarity (%)[a]	Coverage (%)
W144	Fungi	*Aspergillus sydowii* CBS 593.65	100.00	96
W39		*Penicillium panissanguineum* CBS 140989	98.30	95
W78		*Aspergillus aflatoxiformans* CBS 143679	99.66	100
W94		*Aspergillus jensenii* NRRL 58600	98.73	96
W131		*Talaromyces pseudofuniculosus* FMR 15307	99.28	99
W01		*Penicillium ochrochloron* CBS 357.48	99.82	98
W02	Bacteria	*Ensifer adhaerens* strain NBRC 100388	100.00	100
W11		*Paraburkholderia phenoliruptrix* strain AC1100	98.51	100
W13		*Bacillus cereus* ATCC 14579	100.00	100
W20		*Paraburkholderia madseniana* strain RP11	99.36	100
W28		*Pseudomonas alkylphenolica* strain KL28	99.43	100
W37		*Burkholderia stabilis* strain LMG 14294	99.79	100
W46		*Sinomonas humi* strain MUSC 117	98.29	100
W60		*Paraburkholderia lacunae* strain S27	99.01	100
W15		*Priestia aryabhattai* B8W22	100.00	100

[a] ITS or 16S rDNA sequences were used to analyze the fungal or bacterial strains, respectively, against the NCBI database.

Table S2 Predicted genes involved in PE degradation pathway in *A. sydowii* W144 based on its genome sequence.

Gene ID	Protein identity (%)	Enzyme
EVMptg000005lG015730.1	47.54	Multicopper oxidase/ Laccase
EVMptg000004lG001540.1	41.82	
EVMptg000005lG008210.1	36.00	
EVMptg000005lG008460.1	35.51	
EVMptg000009lG002910.1	35.45	
EVMptg000001lG000600.1	34.54	
EVMptg000004lG001590.1	33.33	
EVMptg000002lG001030.1	30.14	
EVMptg000003lG000750.1	29.98	
EVMptg000009lG000410.1	28.57	
EVMptg000001lG024560.1	28.57	
EVMptg000001lG024160.1	28.39	
EVMptg000001lG000600.1	28.25	
EVMptg000007lG007130.1	83.28	Peroxidase
EVMptg000001lG010870.1	52.75	
EVMptg000008lG005920.1	51.72	
EVMptg000001lG012850.1	34.78	
EVMptg000006lG000980.1	31.94	
EVMptg000005lG007990.1	79.31	Hydroxylase
EVMptg000005lG012370.1	53.19	
EVMptg000007lG004210.1	50.00	
EVMptg000007lG011660.1	50.00	
EVMptg000003lG010260.1	50.00	
EVMptg000004lG009400.1	47.06	
EVMptg000004lG002210.1	44.68	
EVMptg000001lG005710.1	42.67	
EVMptg000001lG015900.1	41.33	
EVMptg000005lG007640.1	40.48	
EVMptg000001lG011670.1	40.00	
EVMptg000003lG006700.1	39.58	

EVMptg000007lG015280.1	38.46	
EVMptg000002lG010380.1	37.84	
EVMptg000001lG015080.1	37.50	
EVMptg000007lG005460.1	36.00	
EVMptg000003lG010490.1	35.21	
EVMptg000007lG015180.1	34.54	
EVMptg000009lG003660.1	33.96	
EVMptg000007lG015920.1	32.65	
EVMptg000005lG007070.1	32.47	
EVMptg000003lG007880.1	32.08	
EVMptg000002lG016110.1	32.00	
EVMptg000005lG003760.1	30.77	
EVMptg000002lG014120.1	30.26	
EVMptg000008lG002530.1	29.11	
EVMptg000002lG001520.1	28.77	
EVMptg000008lG003230.1	28.33	
EVMptg000002lG012010.1	93.63	
EVMptg000004lG015850.1	67.98	
EVMptg000002lG001860.1	53.66	
EVMptg000006lG000370.1	45.16	
EVMptg000002lG009950.1	44.48	
EVMptg000009lG004350.1	40.54	
EVMptg000009lG000820.1	37.36	
EVMptg000005lG009870.1	36.91	
EVMptg000005lG018400.1	33.95	Alcohol dehydrogenase
EVMptg000004lG000370.1	33.90	
EVMptg000007lG003130.1	33.68	
EVMptg000003lG007520.1	32.93	
EVMptg000007lG015880.1	32.67	
EVMptg000005lG014020.1	32.23	
EVMptg000008lG008390.1	32.17	
EVMptg000005lG000540.1	31.90	
EVMptg000007lG015870.1	31.76	
EVMptg000004lG014310.1	31.69	

EVMptg000007lG015550.1	31.03	
EVMptg000007lG006470.1	30.88	
EVMptg000008lG001220.1	30.82	
EVMptg000001lG014000.1	30.77	
EVMptg000006lG003100.1	30.36	
EVMptg000005lG020350.1	30.18	
EVMptg000007lG001840.1	30.16	
EVMptg000009lG000970.1	29.92	
EVMptg000002lG016430.1	29.73	
EVMptg000007lG000520.1	29.68	
EVMptg000001lG021110.1	29.60	
EVMptg000001lG015780.1	29.52	
EVMptg000001lG024230.1	29.25	
EVMptg000002lG001700.1	29.09	
EVMptg000005lG016470.1	28.78	
EVMptg000006lG002520.1	28.71	
EVMptg000001lG021510.1	28.70	
EVMptg000003lG001170.1	28.52	
EVMptg000006lG000930.1	28.31	
EVMptg000005lG001810.1	28.11	
EVMptg000004lG000080.1	28.02	
EVMptg000006lG007610.1	28.00	
EVMptg000001lG004880.1	88.26	Aldehyde dehydrogenase
EVMptg000006lG005700.1	66.15	
EVMptg000001lG011560.1	65.04	
EVMptg000007lG000020.1	64.45	
EVMptg000003lG000130.1	56.39	
EVMptg000005lG016480.1	52.23	
EVMptg000005lG013160.1	50.49	
EVMptg000004lG000530.1	49.30	
EVMptg000003lG000520.1	49.00	
EVMptg000006lG000500.1	42.80	
EVMptg000007lG007040.1	41.33	
EVMptg000001lG024680.1	41.33	

EVMptg000007lG002470.1	40.92
EVMptg000002lG014470.1	40.61
EVMptg000006lG007710.1	39.15
EVMptg000004lG016960.1	39.10
EVMptg000001lG022060.1	38.42
EVMptg000002lG007180.1	38.13
EVMptg000002lG000340.1	37.99
EVMptg000004lG000460.1	37.73
EVMptg000007lG000340.1	37.50
EVMptg000008lG003160.1	37.26
EVMptg000004lG012990.1	36.96
EVMptg000001lG022700.1	36.05
EVMptg000001lG007810.1	35.82
EVMptg000016lG000030.1	35.73
EVMptg000006lG002120.1	35.51
EVMptg000002lG006790.1	35.46
EVMptg000009lG003940.1	35.04
EVMptg000007lG005080.1	34.96
EVMptg000008lG007740.1	34.83
EVMptg000002lG000550.1	34.66
EVMptg000009lG001470.1	34.59
EVMptg000004lG000060.1	34.43
EVMptg000001lG022190.1	34.42
EVMptg000001lG022090.1	33.91
EVMptg000005lG000320.1	33.88
EVMptg000007lG002500.1	33.60
EVMptg000005lG018610.1	33.53
EVMptg000001lG009200.1	32.54
EVMptg000002lG016410.1	31.60
EVMptg000004lG014470.1	30.82
EVMptg000001lG012070.1	30.39
EVMptg000001lG023700.1	30.34
EVMptg000008lG002860.1	30.33
EVMptg000005lG000080.1	30.10

EVMptg000006lG003280.1	29.92
EVMptg000005lG000130.1	29.65
EVMptg000003lG012700.1	29.65
EVMptg000007lG009360.1	29.57
EVMptg000007lG013690.1	29.39
EVMptg000005lG013380.1	29.35
EVMptg000004lG011600.1	29.12
EVMptg000009lG000250.1	28.91
EVMptg000004lG006680.1	28.53
EVMptg000004lG002860.1	28.21

Note: The multicopper oxidase from *Aspergillus clavatus* (NCBI accession No. XM_001273715.1), the peroxidase from *Aspergillus oryzae* (NCBI accession No. XM_001826850.3), the hydroxylase from *Aspergillus nomiae* (NCBI accession No. XM_015552083.1), the alcohol dehydrogenase from *Aspergillus nidulans* （NCBI accession No. XM_655542.1） ,and the aldehyde dehydrogenase from *Aspergillus nidulans* (NCBI accession No. XM_677211.1) were aligned against the protein sequences of *A. sydowii* W144. The proteins exhibiting identity more than 28% were shown in this table.

Table S3. Summary of reported PE-degrading microorganisms and their degradation characteristics.

Strain	Source	Polyethylene Material	Degradation Time	Significant Changes	References
Aspergillus sydowii W144	Landfill soil	LDPE film	30 days	Mw decreased by 50.93%; Weight loss of 6.39%	This study
Rhodococcus sp. C-2	Yangtze River estuary seawater	LDPE film (Mw 245, 991)	30 days	Mw decreased by 28.88% (245, 991 decreased to 170, 292)	[1]
Metabacillus niabensis RS120	Arabian Sea seawater	MDPE film (10 μm)	30 days	Crystallinity decreased by 6.23%	[2]
Aneurinibacillus / *Brevibacillus* consortium (IS1, IS3, ISA, ISC)	Wastewater treatment plant, landfill	LDPE and HDPE films/particles	140 days	LDPE film weight loss of 58.21%	[3]
Gordonia sp. C1	20-year landfill	LDPE powder / agricultural film	60 days	Mn decreased by 20.08%	[4]
Bacillus sp. C2	20-year landfill	LDPE powder / agricultural film	60 days	Mn decreased by 51.53%	[4]

Bacillus subtilis ATCC 6051	Korean Culture Collection	LDPE film (3 × 3 cm)	30 days	Weight loss of 3.49%	[5]
Bacillus licheniformis ATCC 14580	Korean Culture Collection	LDPE film (3 × 3 cm)	30 days	Weight loss of 2.83%	[5]
Bacillus cereus SHBF2	Commercial aquaculture pond sediment	PE pellets, UV-sterilized	60 days	Weight loss of 6.87%	[6]
Alcanivorax sp. 24	Marine plastic debris	weathered LDPE (thermo-oxidized at 80 °C for 6 months)	34 days	Mw decreased by 32.0% (122.9 kDa decreased to 83.5 kDa)	[7]
Lysinibacillus xylanilyticus S7-10F + *Aspergillus niger* F1	Landfill soil	LDPE film (UV-pretreated for 25 days)	126 days	Crystallinity decreased	[8]
Pseudomonas knackmussii N1-2	Wastewater treatment plant	LLDPE mulch film (8 μm)	56 days	Weight loss of 5.95%	[9]
Pseudomonas aeruginosa RD1-3	Wastewater treatment plant	LLDPE mulch film (8 μm)	56 days	Weight loss of 3.62%	[9]

Yeast consortium (*Sterigmatomyces halophilus* and others)	Gut of wood-feeding termites	LDPE film (25 μm)	45 days	Mw decreased by 77.9% (234, 000 decreased to 52, 000)	[10]
Aspergillus niger H1C	Plastic-contaminated soil	LDPE strips (partially soaked in vegetable oil)	50 days	Weight loss of 4.25% (~8.5 mg)	[11]
Aspergillus tamarii H6C	Plastic-contaminated soil	LDPE strips	50 days	Weight loss of 3.79% (~9.5 mg)	[11]
Aspergillus ochraceopetaliformis H3C	Plastic-contaminated soil	LDPE strips	50 days	Weight loss of 1.98% (~3.8 mg)	[11]
Aspergillus niger/flavus/oryzae consortium	—[a]	LDPE film	55 days	Weight loss of 26.15% (1.7 mg / 6.5 mg)	[12]
Aspergillus flavus PEDX3	*Galleria mellonella* gut	HDPE microplastic particles (<200 μm), UV-sterilized	28 days	Mw decreased by 59.5% (222, 003 decreased to 89, 801)	[13]
Hypocrea lixii	Decayed wood	LDPE milk-packaging fragments, water-leached (50 °C, 5 days)	35 days	Weight loss of 60%	[14]

Alternaria alternata FB1	Marine sediment (Qingdao)	PE film (0.25 mm), ethanol-sterilized	120 days	Mw decreased by 95% (231, 017 decreased to 11, 959)	[15]
Aspergillus niger (TA3)	Landfill soil	LDPE film (commercial plastic bags, thicknesses of 25 and 40 µm)	45 days	Weight loss of 46.57 ± 0.05%	[16]
Cladosporium basi-inflatum (CPEF-6)	Landfill soil	LDPE film (0.038 mm thick, 3 × 3 cm), thermally treated at 75 °C for 7 days	30 days	Weight loss of 0.43 ± 0.01%	[17]
Lasiodiplodia iranensis	Fungal culture collection laboratory	Commercial PE film (0.0125 mm thick, 4 × 4 cm)	60 days	Mw decreased by 2.65% (82, 025 decreased to 79, 849 Da)	[18]
Arsenicibacter MAG10, MAG21, MAG26	Wastewater treatment plant	PE film (0.020 mm thick, 30 × 30 mm)	28 days	Weight loss of 2.95 ± 0.73%	[19]

[a]no data.

References

1. Rong, Z.; Ding, Z.-H.; Wu, Y.-H.; Xu, X.-W., Degradation of low-density polyethylene by the bacterium *Rhodococcus* sp. C-2 isolated from seawater. *Sci. Total Environ.* **2024,** 907, 167993.
2. Sardar, R. K., Discovery and biodegradation characterization of polyethylene by *Metabacillus niabensis*. *Front. Microbiol.* **2025,** 16:1693690.
3. Skariyachan, S.; Patil, A. A.; Shankar, A.; Manjunath, M.; Bachappanavar, N.; Kiran, S., Enhanced polymer degradation of polyethylene and polypropylene by novel thermophilic consortia of *Brevibacillus* sps. and *Aneurinibacillus* sp. screened from waste management landfills and sewage treatment plants. *Polym. Degrad. Stab.* **2018,** 149, 52-68.
4. Chen, X.; Xue, H.; Jiang, Z.; Zhao, J.; Xu, T.; Su, J.; Li, Z.; Zhou, J.; Dong, W.; Cui, Z., Biodegradation of polyethylene by *Gordonia* sp. C1 and *Bacillus* sp. C2 isolated from landfill. *J. Environ. Chem. Eng.* **2025,** 13, (3), 116443.
5. Yao, Z.; Seong, H. J.; Jang, Y. S., Degradation of low density polyethylene by *Bacillus* species. *Appl. Biol. Chem.* **2022,** 65, (1), 1-9.
6. Hossain, S.; Shukri, Z. N. A.; Waiho, K.; Ibrahim, Y. S.; Kamaruzzan, A. S.; Rahim, A. I. A.; Draman, A. S.; Wahab, W.; Khatoon, H.; Kasan, N. A., Biodegradation of polyethylene (PE), polypropylene (PP), and polystyrene (PS) microplastics by floc-forming bacteria, *Bacillus cereus* strain SHBF2, isolated from a commercial aquafarm. *Environ. Sci. Pollut. Res.* **2024,** 31, (22), 32225-32245.
7. Zadjelovic, V.; Erni-Cassola, G.; Obrador-Viel, T.; Lester, D.; Eley, Y.; Gibson, M. I.; Dorador, C.; Golyshin, P. N.; Black, S.; Wellington, E. M. H.; Christie-Oleza, J. A., A mechanistic understanding of polyethylene biodegradation by the marine bacterium *Alcanivorax*. *J. Hazard. Mater.* **2022,** 436, 129278.

8. Esmaeili, A.; Pourbabaee, A. A.; Alikhani, H. A.; Shabani, F.; Esmaeili, E., Biodegradation of Low-Density Polyethylene (LDPE) by Mixed Culture of *Lysinibacillus xylanilyticus* and *Aspergillus niger* in Soil. *PLoS ONE* **2013,** 8, (9), e71720.
9. Hou, L.; Xi, J.; Liu, J.; Wang, P.; Xu, T.; Liu, T.; Qu, W.; Lin, Y. B., Biodegradability of polyethylene mulching film by two *Pseudomonas* bacteria and their potential degradation mechanism. *Chemosphere* **2022,** 286, 131758.
10. Elsamahy, T.; Sun, J.; Elsilk, S. E.; Ali, S. S., Biodegradation of low-density polyethylene plastic waste by a constructed tri-culture yeast consortium from wood-feeding termite: Degradation mechanism and pathway. *J. Hazard. Mater.* **2023,** 448, 130944.
11. Rojas-Villacorta, W.; Cruz-Noriega, M. D. L.; Otiniano, N. M.; Terrones-Rodríguez, N.; Quiñones-Cerna, C., Biodegradation of low-density polyethylene by native *Aspergillus* strains isolated from plastic-contaminated soil. *Sustainability* **2025,** 17, (20), 8983.
12. Dsouza, G. C.; Sheriff, R. S.; Ullanat, V.; Shrikrishna, A.; Joshi, A. V.; Hiremath, L.; Entoori, K., Fungal biodegradation of low-density polyethylene using consortium of *Aspergillus* species under controlled conditions. *Heliyon* **2021,** 7, (5), e07008.
13. Zhang, J.; Gao, D.; Li, Q.; Zhao, Y.; Li, L.; Lin, H.; Bi, Q.; Zhao, Y., Biodegradation of polyethylene microplastic particles by the fungus *Aspergillus flavus* from the guts of wax moth *Galleria mellonella*. *Sci. Total Environ.* **2020,** 704, 135931.
14. Chaturvedi, M.; Kaur, N.; Alam, S.; Sharma, S., Sustainable approach for degradation of low-density polyethylene plastic waste using ligninolytic white rot fungus. *J. Basic Microbiol.* **2025,** 65, (4), e2400442.
15. Gao, R.; Liu, R.; Sun, C., A marine fungus *Alternaria alternata* FB1 efficiently degrades polyethylene. *J. Hazard. Mater.* **2022,** 431, 128617.
16. Asfaw, T. D.; Muleta, A.; Awlachew, Z. T., Biodegradation of low-density polyethylene (LDPE) bags using fungal isolates from Gondar municipal solid waste disposal soil. *Discov. Environ.* **2026,** 4, (1), 30.

17. Gong, Z.; Jin, L.; Yu, X.; Wang, B.; Hu, S.; Ruan, H.; Sung, Y. J.; Lee, H. G.; Jin, F., Biodegradation of low density polyethylene by the gungus *Cladosporium* sp. recovered from a landfill site. *J. Fungi* **2023,** 9, (6).

18. Okal, E. J.; Zhou, J.; Wu, Y.; Zhong, T.; Tang, Y.; Sun, Z.; Xu, R.; Hu, Y.; Hu, N.; Li, J.; Karunarathna, S. C.; Mortimer, P. E.; Iqbal, S.; Yu, D.; Xu, J.; Gui, H., Unveiling fungal degradation pathways for polyurethane and polyethylene through enrichment cultures and metabolic analysis. *Int. Biodeterior. Biodegrad.* **2025,** 202, 106097.

19. Li, Q.; Li, H.; Tian, L.; Wang, Y.; Ouyang, Z.; Li, L.; Mao, Y., Genomic insights and metabolic pathways of an enriched bacterial community capable of degrading polyethylene. *Environ. Int.* **2025,** 197, 109334.
